# The synergistic effect of Selenium (selenite, –SeO_3_^2−^) dose and irradiance intensity in *Chlorella* cultures

**DOI:** 10.1186/s13568-017-0348-7

**Published:** 2017-03-07

**Authors:** Azadeh Babaei, Karolína Ranglová, Jose R. Malapascua, Jiří Masojídek

**Affiliations:** 1 Laboratory of Algal Biotechnology, Centre ALGATECH, Institute of Microbiology, CZ–37981 Třeboň, Czech Republic; 2 Faculty of Science, University of South Bohemia, CZ-37005 České Budějovice, Czech Republic

**Keywords:** *Chlorella*, Chlorophyll fluorescence, Growth, Irradiance intensity, Photosynthesis, Selenium incorporation

## Abstract

Microalgae are able to metabolize inorganic selenium (Se) to organic forms (e.g. Se-proteins); nevertheless at certain Se concentration culture growth is inhibited. The aim of this work was to confirm the hypothesis that the limit of Se tolerance in *Chlorella* cultures is related to photosynthetic performance, i.e. depends on light intensity. We studied the relation between the dose and irradiance to find the range of Se tolerance in laboratory and outdoor cultures. At low irradiance (250 µmol photons m^−2^ s^−1^), the daily dose of Se below 8.5 mg per g of biomass (<20 µM) partially stimulated the photosynthetic activity (relative electron transport rate) and growth of *Chlorella* cultures (biomass density of ~1.5 g DW L^−1^) compared to the control (no Se added). It was accompanied by substantial Se incorporation to microalgae biomass (~0.5 mg Se g^−1^ DW). When the Se daily dose and level of irradiance were doubled (16 mg Se g^−1^ DW; 500 µmol photons m^−2^ s^−1^), the photosynthetic activity and growth were stimulated for several days and ample incorporation of Se to biomass (7.1 mg g^−1^ DW) was observed. Yet, the same Se daily dose under increased irradiance (750 µmol photons m^−2^ s^−1^) caused the synergistic effect manifested by significant inhibition of photosynthesis, growth and lowered Se incorporation to biomass. In the present experiments Chl fluorescence techniques were used to monitor photosynthetic activity for determination of optimal Se doses in order to achieve efficient incorporation without substantial inhibition of microalgae growth when producing Se-enriched biomass.

**Electronic supplementary material:**

The online version of this article (doi:10.1186/s13568-017-0348-7) contains supplementary material, which is available to authorized users.

## Introduction

Selenium (Se) is a natural trace element that may alternate from an essential micro-nutrient to a toxic compound within a narrow concentration level. Microalgae are able to metabolize inorganic selenium (Se) salts to organic forms as a part of detoxification process, but at certain concentration culture growth becomes inhibited. At nanomolar concentrations Se salts can be beneficial for growth of some microalgae (Li et al. 2003), but further up, at micromolar concentrations may result in a decreased growth rate, photosynthesis inhibition and disturbance to the cell ultrastructure (Morlon et al. 2005; Geoffroy et al. 2007; Fournier et al. 2010; Vítová et al. 2011; Gojkovic et al. 2015). Typical Se content in freshwaters is found in the range of 0.1–2 nM (equivalent to 0.08–0.16 μg Se L^−1^), but much higher concentrations reaching 5 µM (equivalent to 400 µg Se L^−1^) have been observed in contaminated areas (Conde and Sanz Alaejos 1997; Sun et al. 2014). Both selenite (Se^IV^, –SeO_4_^2−^) and selenate (Se^VI^, –SeO_3_^2−^), two major inorganic forms of Se, are toxic to microalgae at higher concentrations (Geoffroy et al. 2007; Morlon et al. 2005) although selenite was found to be more lethal than selenite (Wheeler et al. 1982). These salts are readily incorporated by microalgae, though selenate is accumulated about ten times more efficiently than selenate due to different membrane transporters (Vriens et al. 2016). For nutritional purposes, Se is often supplied in the form of inorganic salts (selenite, selenate), but organically bound selenium in microalgae biomass (Se-proteins) is more beneficial and less toxic to humans and animals and can be used as food and feed supplement (Becker 2007; Doucha et al. 2009; Gojkovic et al. 2015; Kouba et al. 2014; Novoselov et al. 2002).

Microalgae play a crucial role in Se-metabolism due the uptake and biotransformation, and the subsequent transfer upwards into the food chain. Basically, three mechanisms are associated with Se removal by microalgae: (i) biosorption of Se ions on the cell surface, (ii) intracellular uptake of Se ions, and (iii) chemical transformation of Se ions (Neumann et al. 2003; Pardo et al. 2003; Pelah and Ephraim 2005; Oh et al. 2009; Umysová et al. 2009; Gojkovic et al. 2014, 2015). The effects of Se, and its metabolism and bioaccumulation have recently been studied in various groups of microalgae including *Spirulina* (Li et al. 2003; Mane et al. 2013; Pronina et al. 2002), *Scenedesmus* (Umysová et al. 2009), *Chlorella* (Sun et al. 2014; Gojkovic et al. 2014) or *Chlamydomonas* (Geoffroy et al. 2007; Morlon et al. 2005). At low concentration levels, up to 10 mg Se L^−1^, i.e. ~12 µM, cell growth and functioning are not inhibited (Umysová et al. 2009) and microalgae are able to incorporate and transform Se salts to bioavailable organic compounds—proteins containing selenated amino acids, replacing sulphur in methionine and cysteine (for review see ref. Araie and Shiraiwa 2016). Some species also metabolize Se to volatile compounds—dimethylselenide, dimethyldiselenide and dimethylselenenylsulfide (Fan et al. 1997; Larsen et al. 2001; Neumann et al. 2003; Guadayol et al. 2016). Adverse effects of Se on microalgal cultures are usually observed as decrease of population densities and/or growth rates, in proportion to Se concentration in the culture medium. Toxicity to microalgae culture is usually reflected as the corresponding EC50 value. For example, the EC50 values for selenite were found 80 μM in *Chlamydomonas* cultures (Morlon et al. 2005), and 418 μM in *Scenedesmus* (Vítová et al. 2011). At higher doses (above tens of micromoles), Se accumulation may result in inhibited photosynthesis, decreased growth rate, and changes of the cell ultrastructure (Geoffroy et al. 2007; Morlon et al. 2005; Vítová et al. 2011). The uptake of Se in excess to requirements may cause metabolic reactions, possibly some photo-oxidative damage (Chen et al. 2005) that can eventually lead to cell death (Schiavon et al. 2012; Sun et al. 2014).

Fast and sensitive monitoring techniques are employed to detect early stress effects in microalgae cultures. These can be promptly assessed as changes of the photosystem II (PSII) photochemical performance that is reliably determined by chlorophyll (Chl) fluorescence, a sensitive and easy-to-use technique. Recently, we have used fast fluorescence induction kinetics and rapid light-response curves of electron transport activity for detection of various stressors in microalgae cultures (Masojídek et al. 2011a; Malapascua et al. 2014). These techniques reflect the absorption, distribution and utilization efficiency of energy by cells and can indicate Se effect on photosynthetic activity in microalgae cultures (e.g. Geoffroy et al. 2007; Gojkovic et al. 2015).

In this work, we aimed to study the synergistic effect of Se (selenite, SeO_3_^2−^) doses and irradiance intensity on *Chlorella vulgaris* cultures. Changes of photosynthetic performance (measured as Chl fluorescence variables) were investigated in detail in correlation with growth, pigment composition and Se incorporation to biomass. Here, photosynthesis measurements were used in order to control microalgae activity during Se treatment and incorporation. The experiments at various irradiance intensities showed that the rate and extent of Se uptake depends on photosynthetic performance of the culture. In this way suitable doses of Se can be estimated for mass cultivation to produce a bulk of Se-enriched biomass for nutritional purposes.

## Materials and methods

### Organism

The fast-growing microalga *Chlorella vulgaris*, strain *R117* (*CCALA 10258,* Culture Collection of Autotrophic Organisms, Institute of Botany, Třeboň, Czech Republic) was cultivated in a modified inorganic medium (Šetlík et al. 1972; Zachleder and Šetlík 1982), containing the following compounds (in mg L^−1^): KNO_3_, 2021; KH_2_PO_4_, 340; MgSO_4_·7H_2_O, 989; ferric-sodium chelatonate, 18.4; CaCl_2_, 16; H_3_BO_3_, 1.9; MnCl_2_·4H_2_O, 7.3; ZnSO_4_·7H_2_O, 7; CuSO_4_·5H_2_O, 2; CoSO_4_·**7**H_2_O, 1.4; (NH_4_)_6_Mo_7_O_24_, 0.08; and NH_4_VO_3_, 0.06, pH 7.4.

**Fig. 1:**
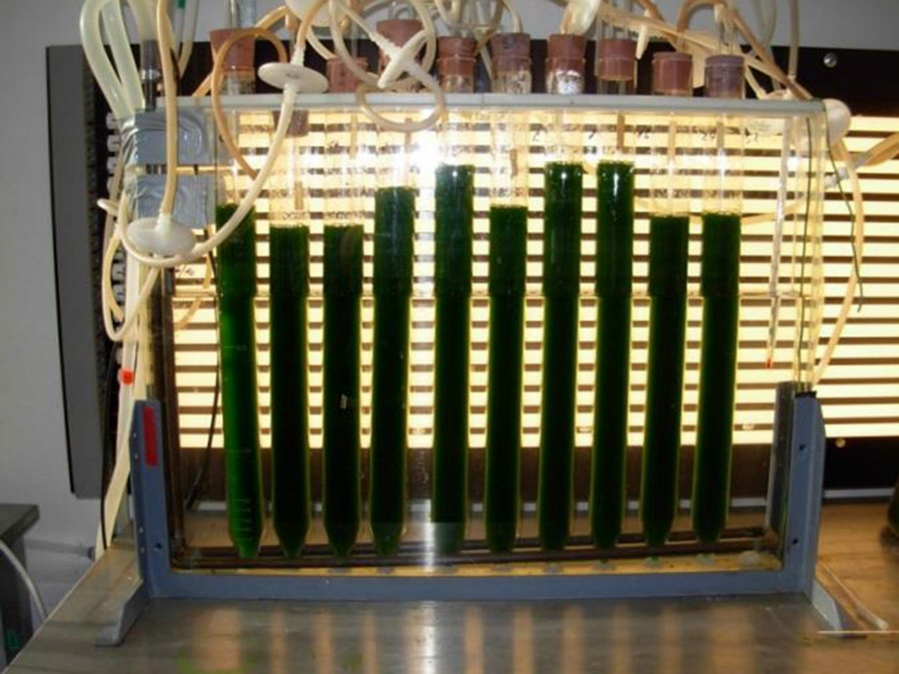
Laboratory cultivation of *Chlorella* in 400 mL vertical columns (mixed by bubbling of air +CO_2_) that were placed in temperature controlled bath with back illumination by high-frequency fluorescent tubes

The cultures were grown in glass columns (working volume 330 mL; internal diameter 35 mm) that were submerged in temperature-controlled water bath (28–29 °C) and mixed by bubbling with air +1.5% CO_2_ (v/v) (Fig. 1). The batch cultures were exposed to continuous illumination between for 4 days. Photosynthetically active radiation (PAR) provided by a horizontal panel of high frequency cool fluorescent tubes (36 W/830 Lumilux, Osram, Germany) was measured directly inside an empty cultivation column to adjust light intensity using a quantum sensor (LI-190SA, cosine-corrected up to 80° angle of incidence) coupled to a light meter (LI-250, Li-Cor, USA).

Outdoor pilot experiment was carried out under ambient irradiance light in thin-layer cascades (area of 220 m^2^ with total cultivation volume of 2200 L) for 7 days in June 2015. More detailed description of the outdoor units and cultivation regime is shown elsewhere (Masojídek et al. 2011b).

### Growth and selenium treatments

Optical density (OD_750_) of the culture was measured at 750 nm using a spectrophotometer as it is approximately linearly related to biomass density (DW). Samples of microalgae cultures were diluted accordingly prior to measurement to keep the absorbance in a linear range. Biomass density was assayed as dry weight (DW) by filtering 5 mL of culture samples on pre-weighed fiberglass filters (GC-50). Then, these were dried in an oven at 105 °C for 3 h, transferred to a desiccator to equilibrate to laboratory temperature and weighed. In all laboratory experiments, well-growing cultures of *Chlorella* were suspended in the fresh medium to the initial biomass density of about 1.5 g/L.

The robust strain R117 of *Chlorella* was selected as model microalga in this work as in preliminary trials it showed tolerance to high doses of selenite (not shown here). In order to select appropriate Se dose in our experiments we have considered the tested concentration range of Se salts in cultivation media that was usually between 0.1 and 80 mg L^−1^ (1–500 μM) (e.g. Bennett 1988; Morlon et al. 2005; Geoffroy et al. 2007; Umysová et al. 2009; Fournier et al. 2010; Gojkovic et al. 2014, 2015).

In one series of experiments, Se was added as a stock solution of sodium selenite (Na_2_SeO_3_) once a day to each cultivation column (250 µmol photons m^−2^ s^−1^) at various concentration (2.5, 8.5 and 25 mg Se per g of starting biomass density; representing final concentrations of about 19, 65 and 190 µmol L^−1^, respectively) to determine effective concentration. In the other series of experiments, various light intensities (250, 500 and 750 µmol photons m^−2^ s^−1^, respectively) were used to study the irradiance-dependent effect of Se treatment (16 mg Se g^−1^ biomass added twice a day as Na_2_SeO_3_; final concentration of about 122 µmol L^−1^). All Se treatments were performed in duplicates. Control cultures were grown in the absence of Se. Distilled water was added to columns before each sampling to compensate volume lost by evaporation.

In outdoor trial Se was added to *Chlorella* cultures in the cascade units in the form of Na_2_SeO_3_ twice a day (at 8 a.m. and 1 p.m.) to keep its concentration of about 20 µmol L^−1^.

### Analysis of selenium content

For Se analysis culture samples (50 mL) were centrifuged and sediments of cells were freeze-dried before analysis. Biomass was mineralized in the microwave decomposition equipment (Milestone 1200 Mega) using nitric acid and hydrogen peroxide. The total amount of Se in biomass was determined using inductively coupled plasma mass spectrometry (ICP-MS, Agilent 7700×) by a commercial company (Healthcare Institute Ltd., Ústí nad Labem, Czech Republic).

### Chlorophyll fluorescence measurements

For Chl fluorescence measurements, samples were taken from the cultures and measured in triplicates (data presented as mean ± SE) at specified daytimes. Before measurement, the cultures were diluted to 0.2–0.3 g DW L^−1^ (corresponding to 5–7 mg Chl L^−1^) with distilled water, dark adapted for 5–10 min and then transferred to a measuring chamber. In this way, we prevented re-absorption problems in a dense culture and provided sufficient illumination in the dark-acclimated samples (with an oxidized plastoquinone pool). Measurements were carried out under well-defined laboratory conditions and ‘light’ exposure history to avoid modifying the photo acclimation state of the cells.

Two Chl fluorescence techniques were used for culture monitoring. While rapid fluorescence induction kinetics provides information on the reduction of the photosynthetic electron transport chain, fluorescence quenching analysis gives information on the balance between photosynthetic electron transport and Calvin-Benson cycle (Malapascua et al. 2014). The fluorescence nomenclature in this paper follows Schreiber et al. (1986) as later elaborated by Kooten and Snel (1990) and Kromkamp and Foster (2003).

#### Rapid light-response curves

Rapid light-response curves (RLCs) of microalgae samples were measured using a pulse-amplitude-modulation fluorometer (PAM-2500, H. Walz, Germany) in a light-protected measuring chamber with mixing (3 mL glass cuvette, light path of 10 mm). A series of stepwise increasing irradiance intensities (red LEDs; 0-2000 μmol photons m^−2^ s^−1^) were applied in 20 s intervals to obtain the ‘steady-state’ fluorescence level (F′) and then a saturating pulse (>10,000 μmol photons m^−2^ s^−1^, 0.6 s duration) was triggered to reach the maximum fluorescence level (F_m_′). At each irradiance intensity, the actual (or effective) photosystem II (PSII) photochemical quantum yield in the light-adapted state, designated as Y_II_ was calculated as (F_m_′–F′)/F_m_′; it represents actual operational ability of photosynthesizing cells to convert light power into chemical energy. The so called relative electron transport rate through PSII, rETR (dimensionless) was calculated as multiplication of Y_II_ by the photosynthetically active radiation E_PAR_, rETR = Y_II_ × E_PAR_ and plotted against E_PAR_ (e.g. Hofstraat et al. 1994; Ralph and Gademann 2005; White et al. 2011). RLCs for ETR were constructed and analyzed to determine important variables. In order to estimate maximum value, rETR_max_, and the value of irradiance saturating photosynthesis, or onset of light saturation (E_k_), the rETR vs. irradiance curves were fitted to the non-linear least-squares regression model by Eilers and Peeters (1988) (PAM Win–3 Software provided by H. Walz), The minimum and maximum fluorescence levels (F_o_, F_m_) were determined using a weak modulated light (<0.15 μmol photons m^−2^ s^−1^, frequency of 0.5–1 kHz) in the dark adapted samples (1st step of RLC; actinic irradiance = 0). The maximum PSII quantum yield was calculated as the ratio of variable to maximum fluorescence, Fv/Fm = (F_m_–F_o_)/F_m_.

#### Fast fluorescence induction kinetics (OJIP-test)

A hand-held fluorometer (AquaPen AP-100, produced by P.S.I. Ltd, Brno, Czech Republic) adapted for liquid samples was used to follow rapid fluorescence induction kinetics (~1 s) in microalgae cultures. Dark-adapted samples (5–10 min) were applied to a 3 mL measuring cuvette (light path of 10 mm) that was mounted in a light-protected holder in front of the detector (adjustable measuring light pulses, ~2.5 µs) while illuminating red LEDs served as high-intensity continuous light source from both sides of the cuvette (up to 3000 µmol photon m^−2^ s^−1^), perpendicular to the fluorescence detector. The fast fluorescence induction kinetics was measured in the time range between 50 µs to 1 s; it started upon illumination (saturating continuous light) of dark-adapted microalgae culture as the signal rises rapidly from the origin (O) to a peak (P) via two infections—J (Vj) and I (Vi) (Strasser and Srivastava 1995; Goltsev et al. 2016). The O point of the fluorescence induction curve is a minimum value (designated as constant fluorescence yield F_o_ at 50 µs) when plastoquinone (PQ) electron acceptors (Q_A_ and Q_B_) of the PSII complex are fully oxidized. It represents the signal emitted from excited Chl-a molecules in the light-harvesting complex II before excitons have migrated to the PSII reaction center. The inflection J occurs after ~2–3 ms of illumination and reflects the dynamic equilibrium (quasi-steady state) between the Q_A_ reduction and its oxidation. The J–I phase (at 30–50 ms) is due to the closure of the remaining centers (further reduction of Q_A_ and various redox states of temporary maximum of Q_A_^−^Q_B_^2−^) and the I–P phase (ends at about 300–500 ms) corresponds to the full reduction of the plastoquinone pool (equivalent to maximum fluorescence level F_m_).

### Pigment analysis

Chlorophyll and total carotenoid contents were determined in 80% acetone by breaking microalgae cells by intensive abrasion with sea sand for 2 min using a vortex mixer. The supernatant containing pigments was collected after centrifugation. The extraction was repeated several times until the pellet was clear of pigments. The absorbance of the combined supernatants of all extraction steps was measured using a high resolution spectrophotometer with a slit width of 0.5 nm (UV 2600 UV–VIS, Shimadzu, Japan) and the concentrations of pigments were calculated according to Wellburn (1994).

### Statistical analysis

Sigma Plot 11.0 was used to determine significant differences between treatments. One-way ANOVA and Post-Hoc test was conducted for comparison of Se treatment to control cultures. *P* values lower than 0.05 were considered to be significantly different. Statistical analysis was only done for pigment contents and Se accumulation in microalgae biomass.

## Results

In one series of laboratory experiments (Trial 1), various concentrations of Se (2.5, 8.5, 25 and 85 mg g^−1^ DW) were applied to *Chlorella* cultures under low irradiance of 250 µmol photons m^−2^ s^−1^ to determine effective Se dose that inhibits photosynthesis and subsequently growth and correlate these variables with Se uptake. In the other series of laboratory experiments (Trial 2) the concentration of 16 mg Se g^−1^ DW was used under various light intensities (250, 500 and 750 µmol photons m^−2^ s^−1^) to study the irradiance-dependent rate of Se uptake. The biomass density at the start of both experiments was 1.2–1.6 g DW L^−1^. Finally, laboratory experimental data were used in outdoor cultivation of *Chlorella* (Trial 3) to produce a bulk of Se-enriched biomass for nutritional purposes.

### Trial 1: changes in *Chlorella* cultures caused by various Selenium concentrations

**Fig. 2:**
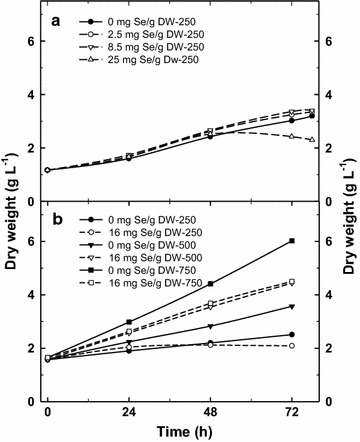
Changes in the biomass density (DW) of the *Chlorella* cultures treated with various Se doses (2.5, 8.5 and 25 mg Se per g of DW): **a** with addition of various Se concentrations at 250 μmol photon m^−2^ s^−1^; **b** with addition of 16 mg g^−1^ selenium exposed to various light intensities (250, 500 and 750 µmol photons m^−2^ s^−1^) during cultivation period of 72 h (control—no Se added)

In this series of experiments the *Chlorella* growth and physiology was studied after the addition of various Se concentration (added as selenite) from low and stimulating (2.5 mg g^−1^ DW) to highly inhibiting concentration (85 mg g^−1^ DW) and compared to the control cultures (no Se addition). The Se concentration of 2.5 and 8.5 mg Se g^−1^ DW caused a slight stimulation of growth during 3 day trial. A sigmoidal course was found with an initial slower phase, followed by acceleration of growth and it started to decelerate after 48 h (Fig. 2a). The results showed that in the control culture (no Se added), the biomass concentration increased by about 2.5 times and it reached stationary phase after 72 h. In the cultures treated with 2.5 and 8.5 mg Se g^−1^ DW, the growth rate was slightly faster. In case of the 25 mg Se g^−1^ DW, the culture grew up to 48 h and then the biomass density started to decrease. The higher concentration of Se (85 mg Se g^−1^ DW) caused a strong inhibition of growth and photosynthetic activity (not shown here).

**Fig. 3:**
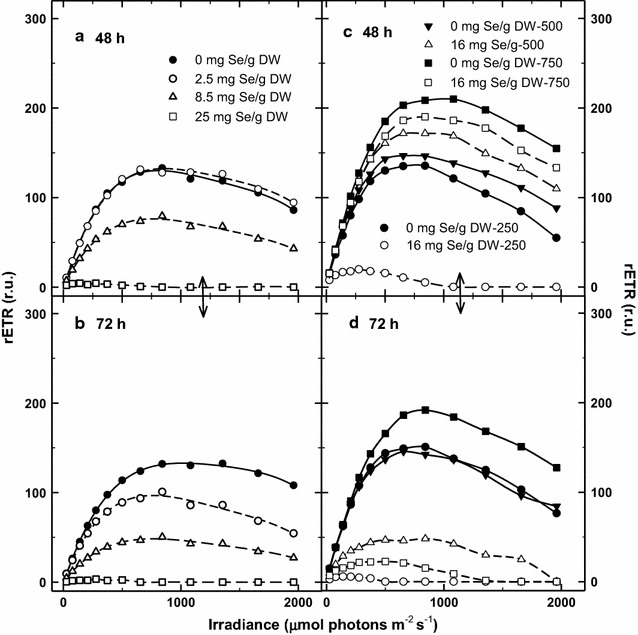
Changes of rapid light-response curves of rETR monitored in the *Chlorella* cultures treated with various Se concentrations (2.5, 8.5 and 25 mg Se per g of DW) at 250 μmol photon m^−2^ s^−1^ after 48 h (**a**) and 72 h (**b**) of experiments. The *Chlorella* cultures were also exposed to various light intensities of 250, 500 and 750 µmol photons m^−2^ s^−1^, respectively in the presence of 16 mg Se g^−1^ DW for 48 h (**c**) and 72 h (**d**) (control—no Se addition)

**Fig. 4:**
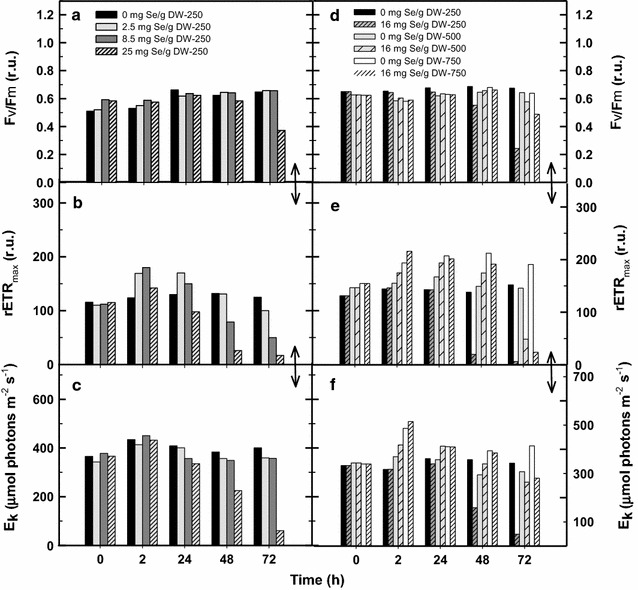
Changes of variables Fv/Fm (**a**) rETR_max_ (**b**) and E_k_ (**c**) were calculated from rapid light-response curves (see Fig. 3) caused by various Se concentrations (2.5, 8.5 and 25 mg Se per g of DW) after 72 h of experiments. The effect of various light intensities of 250, 500 and 750 µmol photons m^−2^ s^−1^ in the presence of exposure to 16 mg g^−1^ selenium on Fv/Fm (**d**) rETR_max_(**e**) and E_k_ (**f**) variables is shown after 72 h. Control means no Se addition

The growth analysis was accompanied by fluorescence measurements of RLCs and OJIP kinetics. Although the growth rate was still similar during first 48 h in the Se treated cultures, we found clear changes in RLCs as compared to the control (Fig. 3). The courses of rETR calculated from RLCs were similar in the control and 2.5 mg Se treated culture, even in the latter there was found slight stimulation after 24 h (Fig. 4). After 48 h, the culture treated with 8.5 mg Se showed about 40% decrease of the rETR activity and the one treated with 25 mg Se g^−1^ DW dropped to 18% (Fig. 3a). Nevertheless, even in the cultures treated with 2.5 and 8.5 mg Se g^−1^ DW, the rETR decreased by 22 and 60%, respectively after 72 h, as compared to the control, most probably due to the onset of Se inhibition. Three variables—maximum PSII quantum yield, Fv/Fm, maximum of relative electron transport rate rETR_max_ and photosynthesis-saturating irradiance E_k_ were calculated from RLCs (Fig. 4a–c). These variables were affected differently as a function of Se concentration. The Fv/Fm values varied a little between 0.61 and 0.66 on Day 1–3, showing that the cultures were in a good physiological state, slightly increasing from Day 1 to Day 3 by 11–27%; only the Fv/Fm values in the culture containing 25 mg Se g^−1^ DW decreased by about 40% on Day 3 (Fig. 4a). The values of rETR in the control culture were rather stable during the trial—between 115 and 130. In the cultures treated with 2.5 and 8.5 mg Se g^−1^ DW, the rETR_max_ values were stimulated up to about 166–177 for the first 24 h, but not in the culture treated with 25 mg Se as it decreased by 20 and 80% on Day 1 and 2, respectively (Fig. 4b). The variable E_k_ corresponding to light saturation is used to characterize the photo acclimation status of microalgae cultures (Serodio et al. 2006). These values showed a decreasing trend after 24 h that probably reflects increasing biomass density and culture acclimation to lower irradiance. A significant drop of the E_k_ values was found only in the cultures treated with 25 mg Se g^−1^ DW; after 48 h of the trial meaning that this culture was strongly inhibited by Se addition (Fig. 4c).

**Fig. 5:**
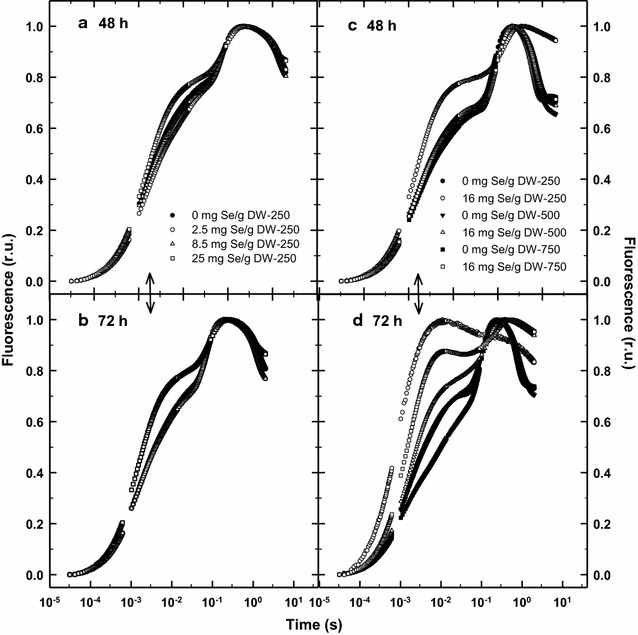
Rapid chlorophyll fluorescence kinetics of *Chlorella* cultures treated with various Se concentrations (2.5, 8.5 and 25 mg Se per g of DW) at 250 μmol photon m^−2^ s^−1^ after 48 h (**a**) and 72 h (**b**) of experiment. The C*hlorella* cultures also were exposed to various light intensities (250, 500 and 750 µmol photons m^−2^ s^−1^, respectively) in the presence of 16 mg Se g^−1^ DW for 48 h (**c**) and 72 h (**d**) (control—no Se addition)

**Fig. 6:**
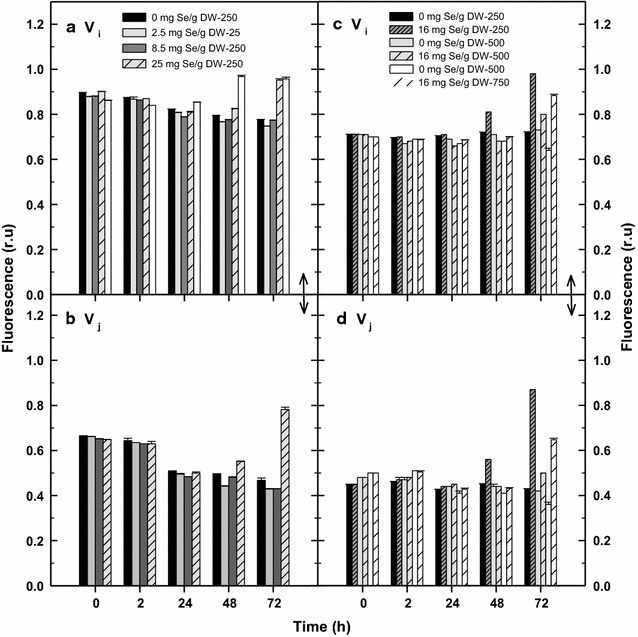
Changes of the Vj (**a**, **c**) and Vi variables (**b**, **d**) calculated from rapid fluorescence induction curves (see Fig. 5) caused by various Se doses (2.5, 8.5 and 25 mg Se per g of DW) after 72 h of experiment (**a**, **b**). The effect of various light intensities of 250, 500 and 750 µmol photons m^−2^ s^−1^ in the presence of exposure to 16 mg g^−1^ selenium on the Vi (**c**) and Vj (**d**) variables shown after 72 h. Control means no Se addition

Rapid fluorescence induction kinetics recorded as OJIP curves in the control and cultures treated with various concentrations of Se indicated inhibitory changes of PSII redox status only at Se concentration of 8.5 and 25 mg Se g^−1^ DW after 48–72 h, respectively. It was manifested by the increased Vj and Vi values in the cultures starting from Day 2 (Figs. 5a, b, 6a, b) which means that the electron transport through the PSII complex was inhibited due to higher reduction of the electron acceptors Q_A_ and Q_B_ and the cultures were not able to utilize the energy input for growth.

**Fig. 7:**
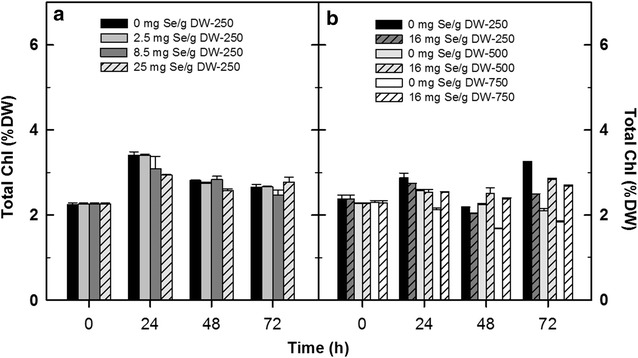
Changes of total Chlorophyll concentrations in *Chlorella* cultures treated with various Se doses (2.5, 8.5 and 25 mg Se per g of DW) exposed to 250 µmol photons m^−2^ s^−1^ after 72 h of experiments (**a**). The effect of various light intensities (250, 500 and 750 µmol photons m^−2^ s^−1^) in the presence of 16 mg Se g^−1^ DW on chlorophyll concentration is shown after 72 h (**b**). *P < 0.05 means significant difference compared to the control (no Se added)

As concerns Chl content in biomass, it was about 2.2% at the start and the maximum values between 3.4 were reached in these laboratory cultures after 24 h; then these decreased to 2.5–2.8 being relatively stable on Days 2 and 3 (Fig. 7a). Higher Se concentration did not significantly affect the total Chl content in the cultures even if the rETR_max_ values were significantly decreased (72 h). The content value of carotenoids was about 0.23% at the start and the maximum value of 0.5% were reached during exposure to 8.5 mg g^−1^ Se after 72 h (data are not shown). This increase in the carotenoids pigments was attributed to the protection provided by carotenoids to chloroplasts against oxidative damage (Schiavon et al. 2012).

**Fig. 8:**
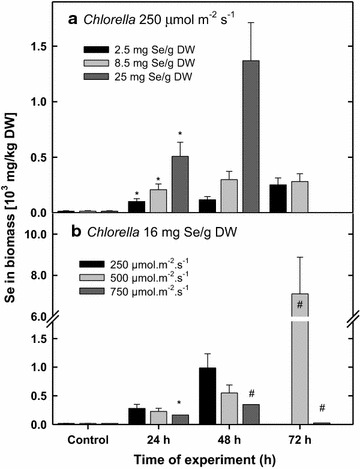
Changes of total Se incorporation to biomass during a 3-day experiment in *Chlorella* cultures exposed to various Se doses (2.5, 8.5 and 25 mg Se per g of DW) under 250 µmol photons m^−2^ s^−1^ (**a**) and various light intensities (250, 500 and 750 µmol photons m^−2^ s^−1^) in the presence of 16 mg Se g^−1^ DW (**b**). *P < 0.05 means significant difference compared to the control (no Se added)

As concerns Se accumulation in *Chlorella* biomass, at low dose (2.5 mg g^−1^ DW) added to the culture, the content was 101 µg Se g^−1^ DW after 24 h that was about 50% of that (208 µg Se g^−1^ DW) found in the culture treated with 8.5 mg g^−1^ DW (Fig. 8a). In the cultures treated with low and medium dose of Se, its accumulation reflected the partial stimulation of rETR_max_ (Fig. 8a vs. Fig. 4b). After 72 h the content of Se in biomass was increased to 251 and 281 µg g^−1^ DW, respectively while about 10 and 55% decrease of rETR, respectively was observed showing the onset of Se inhibition. At high Se dose to the culture (25 mg g^−1^ DW) the course of experiments was different; after 24 and 48 h, the level of Se accumulation was considerably higher—508 and 1370 mg g^−1^ DW, respectively (5–10 times more than in the presence of 2.5 and 8.5 mg Se g^−1^). Here, the inhibitory affect was clearly visible as decrease of rETR_max_ after 24 h (Fig. b). After 72 h Se content in the biomass was too high (not shown here) that we consider it as an artefact, probably due to unspecific adsorption of Se since ETR activity was negligible (Fig. 4b).

The results revealed that at low irradiance intensity (250 µmol photons m^−2^ s^−1^) the doses of Se above 8.5 mg g^−1^ DW were growth-inhibiting. The ‘background’ content of Se in the control cultures was between 15 and 17 µg g^−1^ DW.

### Trial 2: the effect of Selenium on microalgae cultures under various irradiance intensities

Preliminary experiments showed that the limit of Se tolerance is related to photosynthetic activity, i.e. depends on irradiance intensity. Thus, in Trial 2, the effective concentration of 16 mg Se g^−1^ DW was used (~235 µmol L^−1^) to study changes of photosynthetic activity and the extent of Se incorporation into biomass under various light intensities (250, 500 and 750 µmol photons m^−2^ s^−1^). This range of irradiance intensities was assessed from outdoor experiments where we found the average diel irradiance of ~400 μmol photon m^−2^ s^−1^ close to the surface (Masojídek et al. 2011b). The control cultures (no Se added) for each irradiance intensities were set-up in parallel. At low irradiance (250 µmol photons m^−2^ s^−1^), the biomass density increased from 1.57 to 2.5 g L^−1^ during a 3-day trial (Fig. 2b). The rise of light intensity to 500 and 750 µmol photons m^−2^ s^−1^ increased the growth rate of *Chlorella* cultures more than 2 and 4 times, respectively to reach 3.5 and 6 g biomass L^−1^ at the end of the trial. It is important to note that similarly as in Trial 1, a slow-down of growth and photosynthetic activity of the cultures was always accompanied by a release of volatile Se compounds; it indicated that Se was not metabolized.

The courses of growth curves were reflected by RLCs and OJIP kinetics. After 24 h, RLCs measured in the control cultures revealed high rETR values of about 210 at 750 µmol photons m^−2^ s^−1^ while this activity was by 10–20% lower at 500 and 250 µmol photons m^−2^ s^−1^ (Figs. 3c, d, 4e). In the presence of Se, the growth at the medium irradiance (500 µmol photons m^−2^ s^−1^) was stimulated being higher by about 24% after 72 h as compared to the control culture (Fig. 2b). (Interestingly, the course of growth at 500 µmol photons m^−2^ s^−1^ in the Se presence was almost identical as in the control at 750 µmol photons m^−2^ s^−1^). At the medium irradiance rETR was higher in the Se-treated culture after 48 h as compared to the control; the significant inhibition was seen only after 72 h as it dropped to 33% of the initial value (Figs. 3d, 4e). The addition of Se to the low and high irradiance (250 and 750 µmol photons m^−2^ s^−1^) caused a 20–25% inhibition of growth as compared to the control cultures, but we have to consider different final biomass densities (Fig. 2b). Similarly at in the case of RLC, the course of the OJIP curves revealed that the Se-treated cultures at 500 µmol photons m^−2^ s^−1^ were least inhibited as compared to those exposed to 750 and 250 µmol photons m^−2^ s^−1^. At medium irradiance, the Vj and Vi variables were only slightly increased in the presence of Se after 72 h (Fig. 6c, d) while at low and high irradiance these increased significantly indicating a more reduced state of the PSII reaction center, i.e. a block of electron transport.

Chlorophyll content in biomass of the control cultures was about 2.2–2.3% at the start and the maximum values of were reached in low irradiance culture (3.2) while, logically these maxima were 2.1 and 1.8 in the cultures exposed to medium and high irradiance, respectively (Fig. 7b). In the Se–treated cultures, the total Chl content did not changed substantially.

In low-irradiance cultures, Se incorporation was high on Day 2; but after 72 h, similarly as in Trial 2 at high Se dose, the content of Se in biomass was extremely high (not shown here) that can be consider as an artefact, probably due to unspecific adsorption of Se to deprived cells as ETR activity was negligible (Fig. 4d). The highest Se accumulation in biomass (7100 µg g^−1^ DW) was found in *Chlorella* cultures under medium irradiance after 72 h (Fig. 8b) when rETR was still 27% of the maximum activity. As the Se-content of Se in the control cultures was between 15 and 17 µg g^−1^ DW, it means that well-growing *Chlorella* can accumulate Se up to thousand-times in their biomass. At high irradiance the Se incorporation into biomass was low that we consider as the consequence of activity inhibition by the Se+ high irradiance synergism.

### Trial 3: cultivation of outdoor *Chlorella* culture with selenium

In Trials 1 and 2, laboratory *Chlorella* cultures grown under continuous illumination were able to well tolerate certain concentrations of Se (2.5 mg g^−1^ DW, corresponding to 37 μmol L^−1^) without substantial inhibition of growth for 3 days (Figs. 2, 3, 4). On the basis of Trial 1 and 2 and some preliminary outdoor studies (not shown here) we estimated the dose of Se for outdoor longer-term trial test presented here. The aim of this experiment was to produce Se-enriched *Chlorella* biomass in longer run with high amount of Se incorporated into biomass while minimizing growth inhibition and Se losses.

**Fig. 9:**
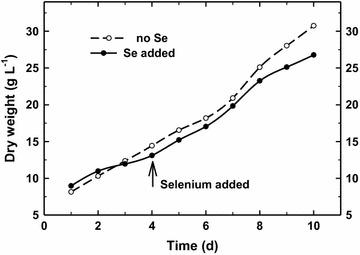
Growth curves of *Chlorella* cultures grown in outdoor thin-layer cascades in the presence (*solid line*) and absence (*dashed line*) of Se during the 10-days trial

The starting biomass density was about 13 g L^−1^ as we took in consideration that light path in outdoor thin-layer cascades was only 6 mm and the trial was carried out at the beginning of June when daily irradiance is relatively high and the diel period is long. In this trial the average concentration of Se was about 20 µmol L^−1^, i.e. total dose of Se was about 0.8 mg g^−1^ biomass supplied during 7 days. This dose was adjusted significantly lower than the lowest dose (2.5 g Se g^−1^ DW) in laboratory Trial 1 that still caused inhibition of rETR after 3 days of experiment and volatile Se compounds were released. In Trial 3 we intended to avoid substantial inhibition of growth. Indeed, in the Se-treated culture was only slightly decreased (5–10%) as compared with the control culture (Fig. 9). No substantial changes of RLC were found in the middle and at the end of the trial (Day 4 and 7) as compared to Day 1 (Additional file 1: Figure S1). The variables rETR_max_ and E_k_ showed the trends of slightly decreasing activity in the Se-treated culture at midday (Table 1) that probably reflected increased biomass density. Changes of the OJIP-curves (Additional file 1: Figure S2) and the derived Vj and Vi variables also indicated only a negligible decrease of photosynthetic activity of the Se-treated culture as compared to the control (Table 1). Nevertheless, at the end of 1-week trial the biomass density was about 26.7 g L^−1^ (about 15% lower than in the control). The total yield of biomass harvest was about 50 kg containing 650 mg Se kg^−1^ DW; about 80% of the added selenite amount was incorporated. We can conclude that we were able to select suitable Se dose that was only mildly inhibiting photosynthetic activity and growth but still the incorporation of Se to biomass was relatively high (compare with Fig. 8). Chl fluorescence was used as fast and suitable technique to monitor doses of Se salts.

**Table 1:** Changes of variables of the maximum PSII quantum yield, F_v_/F_m_, maximum electron transport rate rETR_max_ and the irradiance saturating photosynthesis E_k_ were estimated from rapid light-response curves (Additional file 1: Figure S1) monitored at midday on Day 1, 4 and 7 during a 7-day experiment in the presence and absence of Se

Culture treatment	Fluorescence variables *(r.u.)*	Day 1	Day 4	Day 7
Control (no Se)	*F*_*v*_ */F*_*m*_	0.77	0.75	0.80
	*rETR*_*max*_	461	404	359
	*E*_*k*_	758	714	531
	*V*_*j*_	0.21	0.22	0.21
	*V*_*i*_	0.43	0.43	0.43
Se treated culture	*F*_*v*_ */F*_*m*_	0.74	0.74	0.78
	*rETR*_*max*_	436	380	332
	*E*_*k*_	665	660	625
	*V*_*j*_	0.24	0.26	0.23
	*V*_*i*_	0.46	0.46	0.46

Changes of variables Vj and Vi were calculated from fast fluorescence induction kinetics (Fig. S2) monitored on Day 1, 4 and 7 during a 10-day experiment in the presence and absence of Se. Vj reflects a reduction of Q_A_^−^ (photochemical phase) while Vi is thought to correspond reflect various redox state of temporary maximum of Q_A_^−^Q_B_^2−^

## Discussion

Toxic effect of Se salts is dependent on microalgae species as well as culturing conditions. Thus, prior to production of Se-enriched biomass, specific toxicity analysis for the selected species is required as seen in our trials. As an example published data for the microalga *Chlorella* can be taken for comparison. She can tolerate Se concentrations of up to tens of mg L^−1^ depending on culturing conditions (Pelah and Ephraim 2005; Gojkovic et al. 2014; Sun et al. 2014). It is important to consider that reported laboratory cultures were mostly grown in flasks in rather diluted suspensions exposed to 50–100 µmol photons m^−2^ s^−1^. Not much attention has been paid to the dependence of Se effect at varying irradiance intensities that have been proven as crucial in our trials. Se doses have been usually expressed in mg per liter or molar concentration, but we have to consider biological effect which depends on the ratio between Se concentration and biomass density (g Se per g DW).

Scarce reports can be found describing the physiological effects of Se in detail (added in the form of selenite or selenate) in correlation to biomass productivity and element uptake in microalgae cultures. Here photosynthesis monitoring is essential technique for cultivation control and evaluation of Se tolerance/toxicity in microalgae cultures. When culturing in excessive Se concentrations the early response of microalgae culture is significant decrease of photosynthetic activity monitored as oxygen evolution or Chl fluorescence quenching (e.g. Geoffroy et al. 2007; Gojkovic et al. 2014; Morlon et al. 2005).

In the present experiments with *Chlorella* cultures, a range of Se concentrations (2.5–85 mg L^−1^, i.e. 0.03–1 mM) was used as referred for various microalgae in the literature (for a review see Gojkovic et al. 2015) and we exposed *Chlorella * cultures to higher irradiance intensity, closer to values measured inside the culture outdoors (250–750 µmol photons m^−2^ s^−1^; Masojídek et al. 2011b) at higher biomass densities (~1.5 g L^−1^). The extent of Se effect was dependent on the culture treatment, i.e. Se dose, irradiance intensity and time of exposure. According to our results, *Chlorella* was able to accumulate Se concentration in her cells up to 1000-fold more than it is normally found during cultivation (Fig. 8). This process was, to a certain extent, stimulated by photosynthetic activity (irradiance intensity) until the synergism of increasing irradiance intensity and of Se dose caused photo inhibition (Figs. 3b, c, 4d–f, 5c, d). It is a typical situation described by Lichtenthaler (1988) as *eu*-*stress,* a mild stress activating cell metabolism and increasing physiological activity vs. *dis*–*stress,* having a negative, damaging effect. We have demonstrated that low Se concentration of about 2.5–8.5 mg g^−1^ DW (19–65 µM) partially stimulated photosynthetic activity (rETR) and growth of *Chlorella* for the first 24 h (Fig. 4b). Further on, Se above the tolerable level showed its toxic effect that was indicated by the significantly decreased growth (Fig. 2). Similar trend was also found by Sun et al. (2014) as the growth of *Chlorella* was promoted by Se at lower concentrations. The inhibitory effect of Se salts on photosynthetic yield has been related to ultrastructural changes (Geoffroy et al. 2007; Morlon et al. 2005; Vítová et al. 2009; Schiavon et al. 2016). Chloroplasts are probably the first target of Se cytotoxicity affecting the stroma, thylakoids and pyrenoids as electron microscopy showed the over-accumulation of starch and the formation of dense granules containing Se (Morlon et al. 2005).

As the Se concentration was increased (25 mg g^−1^ DW, i.e. 190 µmol L^−1^), it led to a significant reduction in photosynthetic activity and subsequently growth rate (Figs. 2a, 3a, b). The decrease of photosynthetic activity was accompanied, for some period, by increased Se incorporation to biomass (0.5–1.5 mg g^−1^ DW). This is in agreement with the results obtained with different microalgae species like *Spirulina, Chlamydomonas* or *Chlorella sorokiniana* (Geoffroy et al. 2007; Gojkovic et al. 2014; Li et al. 2003; Morlon et al. 2005). In our experiments growth inhibition at higher Se doses, which resulted in decreased incorporation of Se, was also accompanied by “sulphurous” smell of volatile compounds released during cultivation. At higher Se doses when the culture activity was significantly inhibited (and Se was not incorporated) was this physiological state always accompanied by “sulphurous” smell of volatile Se compounds released during cultivation as mentioned by Guadayol et al. (2016). Se uptake probably employs the enzymatic metabolism resulting in the substitution of some of the sulphur atoms by Se in both free amino acids and proteins as demonstrated by the presence of Se-analogues of the sulphur amino acids during Se accumulation (Li et al. 2003; Araie and Shiraiwa 2016). The higher Se doses can also result in an enhanced accumulation, probably, due to the sorption to cell walls (Pronina et al. 2002) as these consist of variety of extracellular organic compounds such as polysaccharides and proteins that offers a number of active sites capable of binding metal ions (Mane and Bhosle 2012).

When we exposed *Chlorella* cultures to medium light intensities of 500 µmol photons m^−2^ s^−1^, about a 40% stimulation of growth was observed in a 3-day experiment even in the presence of 16 mg Se g^−1^ DW (Fig. 2). However, further increase of light intensity to 750 µmol photons m^−2^ s^−1^ was photo inhibitory, as it was revealed by lowered Fv/Fm and rETR_max_ values (compared to the control), indicating that high irradiance amplified the effect of Se presence on the PSII complex (Fig. 4e).

It is important to note that rETR was more sensitive variable to the Se treatment as compared to the maximum photochemical yield Fv/Fm (Fig. 4). Actually, the effective PSII photochemical yield Y_II_ (used for rETR calculation) was more sensitive to Se treatment as compared to Fv/Fm (Fig. 4a, b; Geoffroy et al. 2007). In any case, we consider the variable rETR more suitable as compared to as Y_II_ as the former takes into account also irradiance intensity E_PAR_ that influence Se incorporation. Based on our Chl fluorescence data—RLCs and OJIP kinetics, changes of Chl fluorescence variables Fv/Fm, rETR_max_, E_k_, Vj and Vi (Figs. 3, 4, 5, 6) we believe that higher Se doses obstruct photosynthetic electron transport between PSII and PSI due to the over reduction of plastoquinone electron carriers. Thus the balance between excitation and electron transfer rate is changed and results in a more reduced state of the PSII reaction center as also reported by Chen et al. (2016). These conclusions in supported by our results as the increase of Vj and Vi (Fig. 6a and c) indicated accumulation of the reduced plastoquinones that cannot transfer electrons further (Strasser et al. 2004). Some authors hypothesize that the presence of Se probably also affects the Cyt b-_6_f complex (Geoffroy et al. 2007; Gojkovic et al. 2015). Because of their chemical similarity, Se can substitute sulphur in an iron–sulphur protein that is a part of the complex. This may eventually lead to disruption of the photosynthetic electron transport chain as Cyt b_6_f plays the role as an electron carrier from PS II to PS I. Eventually, this also indirectly affects the O_2_ evolution rate as the water-splitting complex is also associated with PS II. These are the early photochemical events affecting photosynthesis, supporting the fact that the PSII complex is very sensitive to abiotic stresses (Jajoo et al. 2016), for example the presence of Se in excess.

A better understanding of the overall effect of Se on microalgae has been needed to develop biotechnological processes for the production of Se-enriched biomass containing valuable organic Se–compounds. The main motivation of this work was to study the interplay between the dose and irradiance in order to find the range of Se tolerance in laboratory and outdoor cultures. Therefore, we wished to find fast and suitable methods and key variables in order to optimize doses of Se salts preventing growth inhibition, or even culture loss in large-scale mass cultivation. Monitoring photosynthetic performance via Chl fluorescence diagnostics can give us the early warning to prevent toxic effect (and also release of unpleasant volatile Se compounds) during cultivation. It can also help to follow metabolic pathway of Se in the cells, but it is out of the scope of this work. From a biotechnological point of view, we are able to achieve efficient incorporation of Se by microalgae cells (Se-enriched of biomass) controlled by photosynthesis monitoring without substantial decrease of growth rate.

## Additional file

**Additional file 1: Fig. S1.** Examples of rapid light-response curves of rETR recorded in *Chlorella* cultures on Day 1, 4 and 7 during the outdoor trials. **Fig. S2**. Examples of fast fluorescence induction kinetics (OJIP curves) recorded in *Chlorella* cultures on Day 1, 4 and 7 during the outdoor trials

## Abbreviations

Chl: chlorophyll
DW: dry weight
ETR: electron transport rate
OJIP: fast fluorescence induction curve
PSII: photosystem II
RLC: rapid light curve
Se: selenium
