# Supplementary material for: The synergistic effect of Selenium (selenite, –SeO32−) dose and irradiance intensity in Chlorella cultures
Source: AMB Express. 2017 Mar 7;7:56. doi: 10.1186/s13568-017-0348-7 (PMC5339263; doi:10.1186/s13568-017-0348-7)
Supplement: Supplementary file 1 — Additional file 1: Fig. S1. Examples of rapid light-response curves of rETR recorded in Chlorella cultures on Day 1, 4 and 7 during the outdoor trials. Fig. S2. Examples of fast fluorescence induction kinetics (OJIP curves) recorded in Chlorella cultures on Day 1, 4 and 7 during the outdoor trials [file 13568_2017_348_MOESM1_ESM.docx]

**Submitted to**: *ABM Express*

**Title**: The synergistic effect of Selenium (selenite, ‑SeO_3_^2-^) dose and irradiance intensity in *Chlorella* cultures

**Authors**: Azadeh Babaei^1, *^, Karolína Ranglová^1^, Jose R.Malapascua^1,2^ and Jiří Masojídek^1,2**^

**Affiliations and addresses:**

*^1^Laboratory of Algal Biotechnology, Centre ALGATECH, Institute of Microbiology, CZ‑37981 Třeboň, Czech Republic*

*^2^ Faculty of Science, University of South Bohemia, CZ-37005 České Budějovice, Czech Republic*

* *Permanent address:*  *School of Chemical Engineering, College of Engineering, University of Tehran, Iran*

*^**^*Corresponding author:

e-mail: [masojidek@alga.cz](mailto:masojidek@alga.cz); phone: +420 384 340460 fax: +420 384 340415

**Fig. S1**

**Fig. S1** Examples of rapid light-response curves of relative electron transport rate (rETR) of *Chlorella* cultures recorded on Day 1, 4 and 7 during the outdoor trial

**Fig. S2**

**Fig. S2** Examples of fast fluorescence induction kinetics (OJIP curves) of *Chlorella* cultures recorded on Day 1, 4 and 7 during the outdoor trial
